# Case Report: Malignant Primary Sellar Paraganglioma With Unusual Genetic and Imaging Features

**DOI:** 10.3389/fonc.2021.739255

**Published:** 2021-11-23

**Authors:** Stefan Stojanoski, Henning Bünsow Boldt, Dusko Kozic, Attila Patócs, Márta Korbonits, Milica Medic-Stojanoska, Olivera Casar-Borota

**Affiliations:** ^1^ Faculty of Medicine Novi Sad, University of Novi Sad, Novi Sad, Serbia; ^2^ Center for Diagnostic Imaging, Oncology Institute of Vojvodina, Sremska Kamenica, Serbia; ^3^ Department of Pathology, Odense University Hospital, Odense, Denmark; ^4^ Department of Clinical Research, University of Southern Denmark, Odense, Denmark; ^5^ Hungarian Academy of Sciences and Semmelweis University, HSA-SE “Lendület” Hereditary Endocrine Tumour Research Group, Budapest, Hungary; ^6^ Centre for Endocrinology, William Harvey Research Institute, Barts, United Kingdom; ^7^ The London School of Medicine and Dentistry, Queen Mary University of London, London, United Kingdom; ^8^ Clinic for Endocrinology, Diabetes and Metabolic Diseases, Clinical Center of Vojvodina, Novi Sad, Serbia; ^9^ Department of Immunology, Genetics and Pathology, Uppsala University, Uppsala, Sweden; ^10^ Department of Clinical Pathology, Uppsala University Hospital, Uppsala, Sweden

**Keywords:** malignant sellar paraganglioma, 68Ga-DOTANOC PET/CT, MRI, *ATRX* mutations, *TP53* mutations

## Abstract

**Background:**

Paraganglioma occurs rarely in the sellar/parasellar region. Here, we report a patient with malignant paraganglioma with primary sellar location with unusual genetic and imaging features.

**Case Presentation:**

A 31-year-old male presented with mild hypertension, headache, nausea, and vomiting. A sellar/parasellar tumor mass was revealed by magnetic resonance imaging (MRI), while an endocrine work-up found partial hypopituitarism, suggesting that it was a non-functioning pituitary tumor. Antihypertensive therapy and hormone replacement were initiated. Tumor reduction was achieved with transsphenoidal neurosurgery. However, histological diagnosis was not possible due to extensive tissue necrosis. After 4 years of stable disease, the residual tumor showed re-growth requiring gamma knife radiosurgery. Four years after the radiosurgery, MRI showed a significant tumor progression leading to a second neurosurgery. This time, pathological and immunohistochemical findings revealed paraganglioma. Plasma levels of metanephrine and normetanephrine were normal. A gene sequencing panel performed on DNA extracted from blood excluded germline mutations in 17 susceptibility genes. The patient developed new tumor masses in the neck, and the third surgery was performed. Immunohistochemistry demonstrated lack of ATRX (alpha thalassemia/mental retardation syndrome X-linked) protein in tumor cells, indicating an *ATRX* gene mutation. Molecular genetic analysis performed on tumor DNA revealed a combination of *ATRX* and *TP53* gene abnormalities; this was not previously reported in paraganglioma. MRI and 68Ga-DOTANOC PET/CT revealed the full extent of the disease. Therapy with somatostatin LAR and 177Lu-DOTATATE Peptide Receptor Radionuclide Therapy (PRRT) was initiated.

**Conclusion:**

Although rare, paraganglioma should be considered in the differential diagnosis of sellar/parasellar tumor lesions, even in the absence of typical imaging features. *ATRX* gene mutation in paraganglioma is an early predictor of malignant behavior and a potential novel therapeutic marker when pharmacological therapy targeting mutated ATRX becomes available.

## Introduction

Paragangliomas (PGLs) are rare neuroendocrine tumors originating from chromaffin tissue derived from the neural crest with an incidence of 0.8/100,000 patients/year. They are extra-adrenal tumors arising from the sympathetic and parasympathetic paraganglia. Head and neck PGLs are of parasympathetic origin and only 1%–3% are associated with elevated catecholamine levels in the circulation (1, 2). Besides epinephrine and norepinephrine, the dopamine level may also be increased in up to one-third of patients with head and neck PGL (3). PGLs in the sellar and parasellar region are extremely rare with 31 cases reported so far (4–8), none of them being secreting (4, 6, 9). Their origin has not been fully elucidated; however, it was suggested that they arise from residual aggregates of paraganglionic cells present during fetal and neonatal period along the tympanic or ciliary nerves or branches of the glossopharyngeal nerve within or close to the cavernous sinus (6, 9).

PGLs typically appear as hypointense lesions on T1-weighted MRI and hyperintense on T2-weighted images. Classic MRI “salt and pepper” appearance of head and neck PGLs, composed of flow voids creating low-signal intensity and hemorrhage creating hyperintense regions on both T1- and T2-weighted images (10), has not been reported in sellar/parasellar PGLs, except two cases demonstrating flow voids (6, 11).

Up to 50% of adult and more than 80% of pediatric PGL cases are hereditary tumors (2) usually associated with germline mutations of succinate dehydrogenase (*SDH*) genes *SDHA, SDHB, SDHC, SDHD*, and *SDHAF2* (9, 12). Clinically, hereditary predisposition can be suggested by positive family history, early onset, multiple tumor occurrence, and association with other tumor types (13). The remaining 50% of adults present with somatic mutations in one of the currently susceptible genes for phaeochromocytomas and paragangliomas (14).

PGLs are mostly benign and slow-growing tumors; however, they may grow invasively and metastasize (2). Malignancy of PGLs is defined by the presence of distant metastases, usually affecting bones and lymph nodes (2, 15). Only two cases of metastasizing parasellar PGLs have been reported (11, 16). Recognizing the potential for malignant behavior of PGLs is challenging and impacts the treatment and survival rate in these patients. Although clinical extra-adrenal location, tumor size (larger than 5 cm) and younger age suggest a poor outcome, until today there are no established clinical, genetic, or molecular predictors of malignancy of PGLs at the time of diagnosis (1). Recently, somatic mutations of *ATRX* and telomerase activation have been reported and strongly associated with aggressive and metastatic behavior in PGLs and pheochromocytomas (14, 17, 18).

Here, we present a challenging case of paraganglioma involving the sellar, parasellar, and neck region, with malignant behavior and distant metastases to bones, associated with high-risk molecular genetic pattern and absence of typical imaging features.

## Case Report

**Figure 1:**
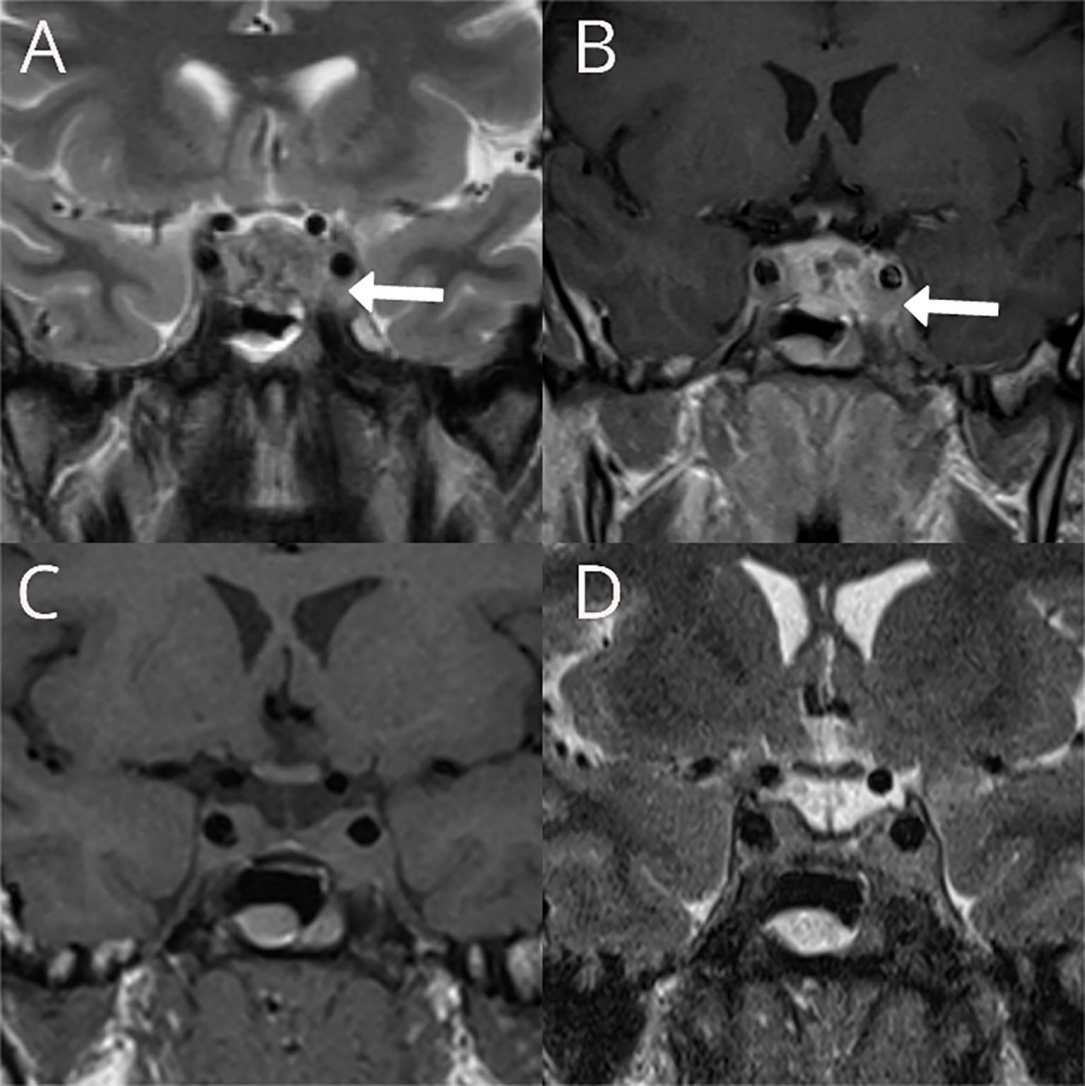
Preoperative T2W coronal **(A)** and T1W coronal image after contrast administration **(B)** showing large sellar mass with bilateral parasellar involvement more prominent on the left, with partial encasement of the internal carotid artery with preserved flow void within the vessel (white arrows). Postoperative T1W coronal **(C)** and T2W **(D)** showing the reduction of the tumor volume in the sella and no significant changes in the parasellar components of the residual tumor.

A 31-year-old male presented with a headache, nausea, and vomiting in November 2008. His family history revealed arterial hypertension in his mother and brother. His father died from liver malignancy, while his paternal uncle died of a brain tumor. Physical examination did not reveal any abnormality, apart from arterial hypertension (150/100 mmHg). Visual fields were normal in both eyes, and no other cranial nerve sensory or motor defects were present. MRI showed the presence of a large sellar mass (16 × 28 × 19 mm) with bilateral parasellar involvement (**Figure 1**). Pituitary hormone assessment showed central hypothyroidism and low prolactin levels (**Table S1**). The clinical diagnosis was of a non-functioning pituitary tumor with partial hypopituitarism. Levothyroxine (50 µg/day) replacement was started. Blood pressure responded well to the ACE inhibitor (Ramipril 1.25 mg/day). Transsphenoidal surgery was performed in February 2009 with partial tumor resection leaving residual tumor in both cavernous sinuses (**Figure 1**). The histopathological examination revealed only necrotic material. Postoperative assessment showed additional gonadotroph and growth hormone deficiency (**Table S1**) and testosterone enanthate (250 mg/month) was initiated. Over the next 4 years, annual MRIs showed a stable residual tumor. In 2013, the patient developed a severe headache, and a pituitary MRI revealed an increase in the residual tumor volume with the same pattern of growth. In November 2013, the patient underwent gamma knife radiosurgery with a dose of 12 Gy to the 50% margin.

Two years later, he developed panhypopituitarism (**Table S1**). He had normal visual fields. Hydrocortisone was added to the replacement therapy (10 mg on waking up and 5 mg late in the afternoon). An MRI follow-up showed mild reduction of the size of the residual tumor.

**Figure 2:**
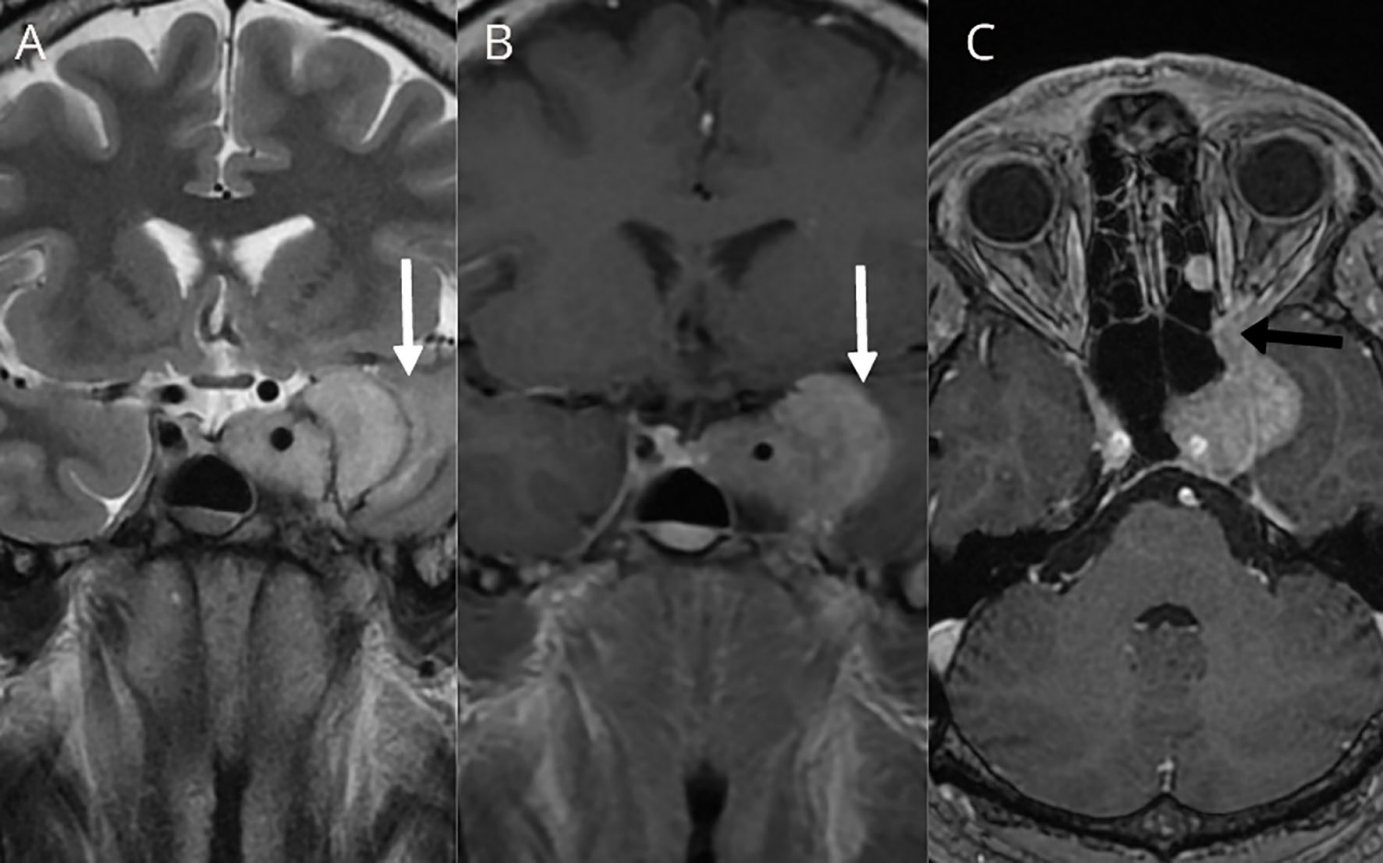
T2W coronal **(A)**, T1W coronal **(B)**, and T1W axial **(C)** after contrast administration showing size increase of the left cavernous sinus residual tumor with mass effect to the left temporal lobe and signs of subcortical vasogenic edema were evident (white arrows). Tumor extension to the left orbit through the orbital fissure was documented (black arrow).

**Figure 3:**
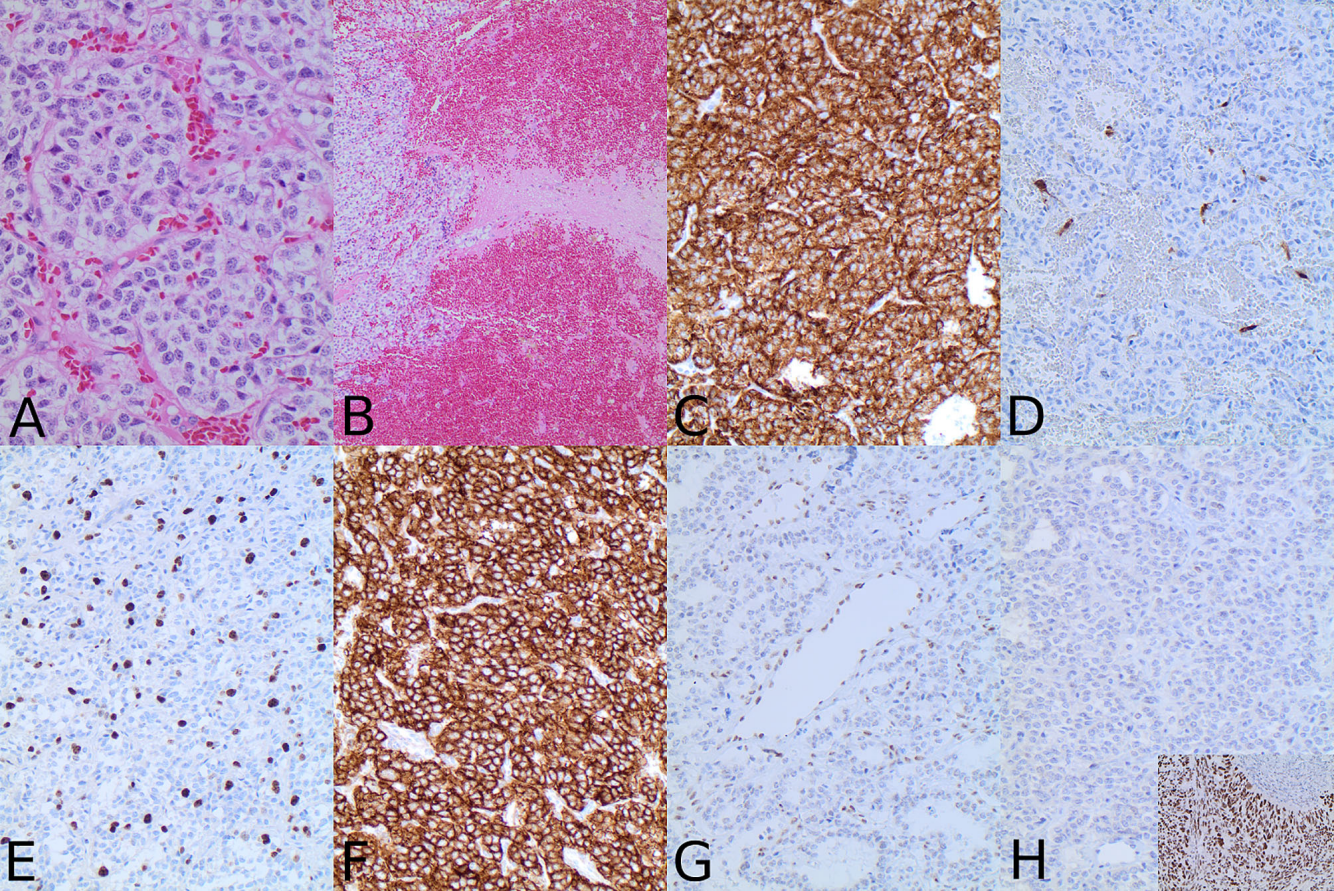
Hematoxylin–eosin staining revealed tumor composed of lobuli of uniform tumor cells surrounded by fibrous septa **(A)** with the evidence of invasion into surrounding brain parenchyma **(B)**. Immunohistochemical analysis with synaptophysin **(C)** confirmed the neuroendocrine nature of the tumor and the presence of S100-positive sustentacular cells **(D)** was typical for paraganglioma. Cell proliferation was increased with Ki67 index reaching 10% **(E)**. Tumor cells were strongly positive for SSTR2A **(F)**. ATRX was preserved in endothelial cells; however, there was no ATRX nuclear immunolabeling in the tumor cells **(G)**. TP53 was negative **(H)**. Insert in H shows TP53 expression in the positive control tissue from a p53 mutated carcinoma (magnification is 200× for all microphotographs except **(A)** that has 400× and **(B)** that has 100×).

In November 2017, the patient was admitted to the emergency room due to loss of consciousness, severe headaches, especially in the left retrobulbar area, nausea, and vomiting. The MRI showed a significant tumor progression (from 22 × 23 × 12 mm to 42 × 35 × 27 mm in 3 years) with tumor extension to the left orbit through the inferior orbital fissure (**Figure 2**). A transcranial surgery was performed with a partial tumor resection. Histopathological examination demonstrated tumor tissue composed of groups of uniform round to oval tumor cells with finely granulated chromatin surrounded by delicate fibrous septa, features suggestive for paraganglioma. Neuroendocrine character of the tumor cells was confirmed by positivity for synaptophysin and chromogranin A. S100-positive sustentacular cells were scattered in the interlobular interstitium. Mitoses were present (fewer than 5 per 10 high power field) and Ki67 proliferative index reached 10% in multiple foci. There were signs of tumor invasion into surrounding brain tissue (**Figures 3A–E**).

Plasma free metanephrine and normetanephrine were within the normal range (**Table S1**). Assessment of the plasma level of dopamine metabolite 3-methoxythyramine was not determined since this analysis is not a routine at our center. Initially, 68Ga-DOTANOC, which is the most sensitive method to detect bone deposits of paraganglioma (19), was not performed. Thus, the presence of the distant metastases early during the clinical course could not be excluded.

The patient’s relatively young age and the aggressive course of the disease prompted the genetic testing. However, next-generation sequencing (NGS) from blood sample showed no germline mutations in *SDHA, SDHB, SDHC, SDHD, SDHAF2, TMEM127, MAX, VHL, RET, NF1, GOT2, MDH2, EGLN, EPAS, SLC25A11, MAX*, and *FH* genes.

**Figure 4:**
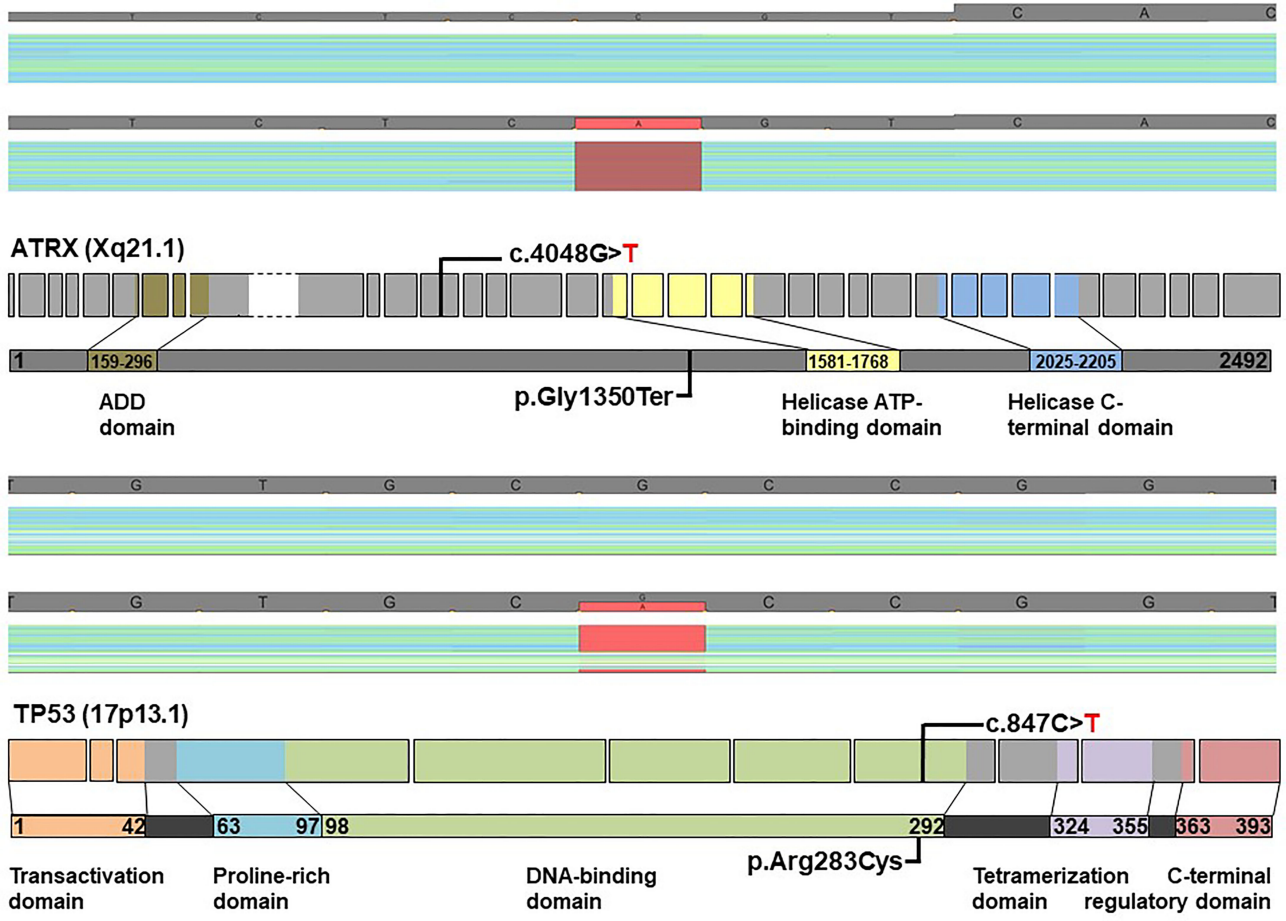
*ATRX* and *TP53* mutations in a patient with metastatic primary sellar paraganglioma. NGS sequencing using a panel with 20 genes commonly mutated in gliomas identified two tumor suppressor mutations: a nonsense mutation in *ATRX* and missense mutation in *TP53* with frequencies of 85% and 50%, respectively. Coverage and pile-up of sequencing raw data visualized using a genome browser depict the mutations in red (lower panels; minus orientation), and healthy donor controls (upper panels) were included for comparison. Schematic diagrams show *ATRX* and *TP53* exon structures with positions of mutations indicated. Only exons containing coding sequence were included (*ATRX*: ex. 1 through ex. 35; *TP53*: ex. 2 through ex. 11). The large exon 9 of *ATRX*, depicted with stippled lines, is compressed for clarity. Protein domains are highlighted in light colors (ADD: ATRX-DNMT3-DNMT3L). The *ATRX* c.4048G>T mutation results in pre-termination of ATRX at G1350 in the central portion of the 2492-residue protein, whereas the c.847C>T mutation of *TP53* causes an R283C substitution in the C-terminus of the DNA binding domain.

The patient was well until January 2019, when left-sided headaches became severe, and tumefaction in the left submandibular region was palpable. The MRI of the neck revealed a tumor mass in the left submandibular region as well as a smaller lesion in the ipsilateral masticator space (**Figure S1**). A nodular soft tissue lesion in the body of the second lumbar vertebra consistent with metastasis was detected on the MRI performed due to back pain (**Figure S1**). The patient underwent stereotactic radiotherapy of the sellar region with the total dose of 48 Gy/24 fractions in May 2019 and posttreatment sellar MRI showed a necrotic transformation of the residual tumor in the left cavernous sinus, while the orbital component remained unchanged. A submandibular lesion was removed. The histopathological findings were principally the same as in the sellar tumor. Immunohistochemical analyses performed according to standard protocols at the Department of Clinical Pathology, Uppsala University Hospital showed strong expression of somatostatin receptors (SSTR) type 2A (**Figure 3F**) and 3 in all tumor cells, whereas SSTR1 and SSTR5 were negative. Additional immunohistochemical analysis with ATRX antibody (HPA001906, Atlas Antibodies; dilution 1:100; incubation time 20 min) demonstrated the lack of immunolabeling in the tumor cells with preserved staining in non-neoplastic cells, strongly suggesting an *ATRX* gene mutation (**Figure 3G**). ATRX immunolabeling was negative both in the specimen from the primary sellar tumor from 2017 and in the specimen from submandibular tumor deposit from 2019. In order to confirm the *ATRX* gene mutation, a molecular genetic analysis was performed using NGS panel covering 20 genes related to CNS malignancies on DNA extracted from formalin-fixed paraffin-embedded tumor tissue from the submandibular tumor lesion. In concordance with lack of ATRX immunolabeling in tumor cells, a nonsense mutation in *ATRX* was detected in exon 12 causing pre-termination at Gly-1350 in the central portion of the ATRX protein preceding the helicase ATP-binding and C-terminal domains. An additional *TP53* p.Arg283Cys missense variant localized in the region encoding the C-terminus of the central DNA-binding domain was also identified. The sample was wild-type with respect to hotspot mutations in *IDH1*, *IDH2*, and *TERT* promoter. No obvious copy number variation (CNV) could be detected (**Figure 4**). Following the molecular genetic finding of a *TP53* variant, immunohistochemical analysis of TP53 (DAKO, monoclonal antibody, clone D0-7, catalogue number GA616; ready-to-use; incubation time 20 min) was performed showing a negative result (**Figure 3H**).

A follow-up MRI in August 2019 revealed an increase in the volume of the masticator lesion and an increased volume of the lumbar metastasis. In December 2019, 68Ga-DOTANOC PET/CT was performed (application 191 MBq) and showed the full extent of the disease (**Figure S2**). Octreotide LAR was initiated LAR (30 mg i.m. every 28 days). Based on the results of 68Ga-DOTANOC PET/CT, Karnofsky performance status (80%), and an increased Ki67 index (10%), we decided to perform Peptide Receptor Radionuclide Therapy (PRRT) with 177Lu-DOTATATE. We performed four out of six planned cycles of the therapy with a dose of 5.55 GBq each following 1-month suspension of octreotide before treatment. During the follow-up, the patient responded well to the treatment; he no longer had headaches and lower back pain, and submandibular swelling has resolved. The follow-up CT, MR, and 68Ga-DOTANOC examinations have been scheduled; however, the patient developed acute calculous cholecystitis that required emergent surgery and resulted in the delayed control examinations.

## Discussion

Here, we report a patient with a primary, aggressive, locally invasive sellar and parasellar paraganglioma with distant bone metastases, lack of typical PGL imaging features, lack of identifiable germline mutation, and a novel molecular genetic profile in the tumor. We draw attention to diagnostic difficulties and underline the need for correlation of clinical findings and family history with radiological, histological, and genetic features in order to identify risk for aggressive behavior and offer the best management to the patients with malignant parasellar paragangliomas.

Unusual sellar/parasellar location of PGLs in combination with the absence of catecholamine hypersecretion and lack of typical MRI characteristics may lead to preoperative misdiagnosis of a sellar/parasellar PGL as pituitary adenoma (20). This was the case in our patient: the tumor initially demonstrated an indolent clinical course with symptoms related to partial hypopituitarism and mild arterial hypertension. MRI features were not characteristic for PGL, except the presence of flow voids that have been previously described (6, 11). All this resulted in preoperative misdiagnosis of the tumor as a non-functioning pituitary adenoma. Unfortunately, histopathological diagnostic was inconclusive due to extensive necrosis and lack of representative tumor tissue.

Although young age at onset in our patient was suggestive for a hereditary tumor, we could not detect any germline mutations by NGS analysis covering 17 known predisposing genes, including all *SDH* genes. After an initial relatively indolent phase, the clinical course in our patient became unusually aggressive with periods of rapid tumor growth, invasion into multiple anatomical compartments, and development of distant metastases. Unfortunately, there are no reliable prognostic markers for early detection of aggressive PGL. Recently, *ATRX* gene mutations were reported in aggressive and malignant PGLs enabling postoperative identification of potentially aggressive cases (18). Immunohistochemistry with antibody towards ATRX results in the lack of the immunostaining in the ATRX-mutated cases and should be performed in all cases in order to identify patients with potentially aggressive PGLs.

In our patient, an immunohistochemical analysis revealed lack of ATRX protein in tumor cells in the specimens from the two last surgeries. A novel nonsense *ATRX* mutation (p.Gly1350Ter) was confirmed by NGS panel performed on DNA extracted from the tumor tissue removed from the submandibular region during the last surgery. Unfortunately, we were not able to explore *ATRX* gene mutational status in the specimen from the first surgery due to necrosis in the tumor tissue. Thus, we cannot make any conclusion whether the *ATRX* mutation was an early event predisposing from the beginning to malignant tumor behavior or whether the mutation developed later on contributing to the malignant progression. However, previous reports on *ATRX* mutations in PGLs (18) and also pituitary neuroendocrine tumors (21) indicate that *ATRX* gene mutation is an early event driving metastatic potential in the ATRX-mutated tumors and can be identified in the primary specimens even years before detection of the metastases. An immunohistochemical analysis with antibody toward ATRX should thus be part of routine pathological work-up in order to identify patients at risk for malignant metastatic disease. *ATRX* gene mutations have been reported in other types of neuroendocrine tumors such as pancreatic NETs (22), aggressive pituitary neuroendocrine tumors (21), and pheochromocytomas (23). The *ATRX* p.Gly1350Ter nonsense mutation has not been previously reported according to search in public database.

The inactivating mutations in *ATRX* are distributed along the coding sequence of this large gene, and accumulation in hotspot regions has not been observed so far. Yet, an *ATRX* p.Gly1350fs mutation also affecting codon 1350 was recently reported (21). The identification by NGS of the *ATRX* mutation in tumor cells are in concordance with the negative ATRX immunostaining as pre-termination of ATRX protein at position 1350 likely results in lack of ATRX expression and in ATRX protein malfunction in the tumor cells. This is supported by pathogenicity assessment using databases (varsome, COSMIC, oncoKB, JAX CKB, dbsnp, and ClinVar), based on the general concept that pre-termination of tumor suppressor genes may cause loss of function. Concurrent somatic mutations in *IDH1* and *ATRX* were noticed in a rare case of sporadic PGL, but the present case was wild-type with respect to hotspot mutations in IDH1 and IDH2 (24).

NGS also showed a missense variant in TP53 translating into a substitution of Arg283 to cysteine. The TP53 p.Arg283Cys variant is rated as a “variant of uncertain significance” (VUS) by the ClinVar database. The functional implication of the detected *TP53* R283C variant needs further investigation. However, the presence of this variant in our case of malignant PGLs may support its oncogenic character. Although *TP53* gene mutations have been sporadically reported in PGL (14, 25, 26) and aggressive PGLs frequently presented with concurring mutations (27, 28), a combination of *ATRX* and *TP53* mutation is so far unique. This co-occurrence has recently been described in aggressive pituitary neuroendocrine tumors and pituitary carcinomas (23). We identified the presence of *TP53* p.Arg283Cys with a frequency of 50% in our patient; however, our NGS approach cannot distinguish somatic from germline mutations because a blood sample from the patient was not included in the sequencing analysis. As both genes are strongly associated with malignant tumors, it is difficult to speculate whether these mutations drive aggressive behavior separately or in combination. The molecular genetic findings give rationale for performing immunohistochemistry with antibodies towards ATRX and p53 in all sellar/parasellar paragangliomas. While ATRX immunohistochemistry is reliable in detection of *ATRX* gene mutations, TP53 immunohistochemistry may be difficult to interpret. Immunohistochemistry for TP53 was negative in our case in the same specimen where we demonstrated *TP53* variant in the tumor cells. Overexpression and distinct TP53 staining are usually seen in a significant proportion of cells in *TP53*-mutated tumors. However, TP53 immunohistochemistry does not always correlate with mutational status of the *TP53* gene. In all cases with the mutations suspected by using immunohistochemistry, *ATRX* and *TP53* mutations should be confirmed by using molecular genetic analysis.

In our patient, the disease could not be controlled despite complex and aggressive therapeutic approach including repeated surgeries, gamma-knife and conventional radiotherapy. A high expression of SSTR2A in tumor cells demonstrated on the basis of both high uptake during 68Ga-DOTANOC PET/CT work-up and immunohistochemical expression of the receptor led us to anticipate that the patient may respond well to somatostatin analogues (SA) (29) and PRRT treatment. PRRT with 177Lu-DOTATATE represents a treatment of choice for metastatic PGLs with high SSTR2 expression (30).

*ATRX* gene is an attractive potential therapeutic target and there are intensive attempts to develop pharmacological therapy for ATRX-mutated tumors (28). When pharmacological therapy targeting mutated ATRX becomes available, patients with confirmed ATRX-mutated paragangliomas will be candidates for the treatment.

In summary, we present a case of an aggressive, metastasizing sellar and parasellar paraganglioma with lack of characteristic MRI imaging features and a somatic loss-of-function *ATRX* gene mutation and a variant of suspect oncogenic potential in *TP53* gene. MRI imaging alone is not specific enough for precise characterization of sellar/parasellar PGLs; therefore, 68Ga-DOTA-peptides PET/CT must be included in the diagnostic algorithm of PGLs, initially for staging and ruling out metastases, and during a follow-up for revealing the full extent of the disease (31–33). The findings provide rationale for immunohistochemical and molecular genetic analysis of *ATRX* and *TP53* in sellar paragangliomas in order to detect the tumors with malignant potential in an early postoperative phase. Moreover, a mutated ATRX is a potential therapeutic target in ATRX-mutated aggressive paragangliomas. Although we could not identify in our patient germline mutations in any of the genes involved in familial paraganglioma, genetic testing should be strongly recommended, especially in patients with confirmed or suspected metastatic disease.

## Data Availability Statement

The original contributions presented in the study are included in the article/**Supplementary Material**. Further inquiries can be directed to the corresponding author.

## Ethics Statement

The studies involving human participants were reviewed and approved by University of Novi Sad, Faculty of Medicine. The patients/participants provided their written informed consent to participate in this study.

## Author Contributions

SS: Radiological investigation and follow-up, writing original draft preparation, and reviewing and editing. HB: molecular genetic examination and validation. DK: radiological supervision and follow-up, and validation of radiological data. AP: genetic analyses and validation. MK: validation and supervision. MM-S: endocrinological examination, writing—reviewing and editing, management and follow-up of the patient, conceptualization, and supervision. OC-B: pathological and immunohistochemical examination, writing—reviewing and editing, and supervision. All authors contributed to the article and approved the submitted version.

## Funding

OC-B was supported by the Swedish Cancer Society (grant 19 0157 Fk) and by the grant from the Swedish state under the agreement between the Swedish government and the county councils (grant number ALF-912341).

## Conflict of Interest

The authors declare that the research was conducted in the absence of any commercial or financial relationships that could be construed as a potential conflict of interest.

## Publisher’s Note

All claims expressed in this article are solely those of the authors and do not necessarily represent those of their affiliated organizations, or those of the publisher, the editors and the reviewers. Any product that may be evaluated in this article, or claim that may be made by its manufacturer, is not guaranteed or endorsed by the publisher.

## References

[1] Nölting S Ullrich M Pietzsch J Ziegler CG Eisenhofer G Grossman A. Current Management of Pheochromocytoma/Paraganglioma: A Guide for the Practicing Clinician in the Era of Precision Medicine. Cancers (2019) 11:1505. doi: 10.3390/cancers11101505 PMC6827093 31597347

[2] Lam AKY. Update on Adrenal Tumours in 2017 World Health Organization (WHO) of Endocrine Tumours. Endocr Pathol (2017) 28(3):213–27. doi: 10.1007/s12022-017-9484-5 28477311

[3] Van Der Horst-Schrivers AN Osinga TE Kema IP van der Laan BF Dullaart RP. Dopamine Excess in Patients With Head and Neck Paragangliomas. Anticancer Res (2010) 30(12):5153–8. 21187504

[4] Lyne SB Polster SP Fidai S Pytel P Yamini B. Primary Sellar Paraganglioma: Case Report With Literature Review and Immunohistochemistry Resource. World Neurosurg (2019) 125:32–6. doi: 10.1016/j.wneu.2019.01.094 30703592

[5] Singh S Kumar A Mehrotra A Rao RN Behari S. Nonsecretory Paraganglioma in Cavernous Sinus Masquerading as Meningioma. World Neurosurg (2019) 126:399–404. doi: 10.1016/j.wneu.2019.02.111 30831293

[6] Naggara O Varlet P Page P Oppenheim C Meder JF. Suprasellar Paraganglioma: A Case Report and Review of the Literature. Neuroradiology (2005) 47:753–7. doi: 10.1007/s00234-005-1422-4 16047139

[7] Schueth EA Martinez DC Kulwin CG Bonnin JM Payner TD Ting JY. Recurrent Primary Intrasellar Paraganglioma. Case Rep Otolaryngol (2020) 2020:2580160. doi: 10.1155/2020/2580160 32685227 PMC7336227

[8] Vasoya P Aryan S Thakar S Sivaraju L Ghosal N Hegde AS. Sellar-Suprasellar Paraganglioma: Report of 2 Cases and Review of Literature. World Neurosurg (2020) 140:293–300. doi: 10.1016/j.wneu.2020.04.157 32413561

[9] Manojlovic-Gacic E Rostami E Karavitaki N Casar-Borota O. Histopathology of Parasellar Neoplasms. Neuroendocrinology (2020) 110:740–52. doi: 10.1159/000507084 PMC7490502 32155632

[10] Woolen S Gemmete JJ. Paragangliomas of the Head and Neck. Neuroimaging Clin N Am (2016) 26:259–78. doi: 10.1016/j.nic.2015.12.005 27154608

[11] Sinha S Sharma MC Sharma BS. Malignant Paraganglioma of the Sellar Region Mimicking a Pituitary Macroadenoma. J Clin Neurosci (2008) 15:937–9. doi: 10.1016/j.jocn.2007.03.029 18482839

[12] Hao HX Khalimonchuk O Schraders M Dephoure N Bayley JP Kunst H. SDH5, a Gene Required for Flavination of Succinate Dehydrogenase, Is Mutated in Paraganglioma. Science (2009) 325:1139–42. doi: 10.1126/science.1175689 PMC3881419 19628817

[13] Neumann HPH Young WF Jr Eng C. Pheochromocytoma and Paraganglioma. N Engl J Med (2019) 381:552–65. doi: 10.1056/NEJMra1806651 31390501

[14] Fishbein L Leshchiner I Walter V Danilova L Robertson AG Johnson AR. Comprehensive Molecular Characterization of Pheochromocytoma and Paraganglioma. Cancer Cell (2017) 31:181–93. doi: 10.1016/j.ccell.2017.01.001 PMC5643159 28162975

[15] Gunawardane PTK Grossman A. Phaeochromocytoma and Paraganglioma. Adv Exp Med Biol (2017) 956:239–59. doi: 10.1007/5584_2016_76 27888488

[16] Haresh KP Prabhakar R Anand Rajan KD Sharma DN Julka PK Rath GK. A Rare Case of Paraganglioma of the Sella With Bone Metastases. Pituitary (2009) 12:276–9. doi: 10.1007/s11102-008-0099-1 18320326

[17] Fishbein L Khare S Wubbenhorst B DeSloover D D’Andrea K Merrill S. Whole- Exome Sequencing Identifies Somatic ATRX Mutations in Pheochromocytomas and Paragangliomas. Nat Commun (2015) 6:6140. doi: 10.1038/ncomms7140 25608029 PMC4302757

[18] Job S Draskovic I Burnichon N Buffet A Cros J Lépine C. Telomerase Activation and ATRX Mutations Are Independent Risk Factors for Metastatic Pheochromocytoma and Paraganglioma. Clin Cancer Res (2019) 25:760–70. doi: 10.1158/1078-0432.CCR-18-0139 30301828

[19] Jha A Patel M Saboury B Millo C Ling A Shah R. Superiority of 68Ga-DOTATATE PET/CT Compared to 18F-FDG PET/CT and MRI of the Spine in the Detection of Spinal Bone Metastases in Metastatic Pheochromocytoma and/or Paraganglioma. A Meeting Report. J Nucl Med (2020) 61(supplement 1):125.

[20] Pepe S Korbonits M Iacovazzo D. Germline and Mosaic Mutations Causing Pituitary Tumours: Genetic and Molecular Aspects. J Endocrinol (2019) 240:R21–45. doi: 10.1530/JOE-18-0446 30530903

[21] Casar-Borota O Boldt HB Engström BE Andersen MS Baussart B Bengtsson D. Corticotroph Aggressive Pituitary Tumors and Carcinomas Frequently Harbor ATRX Mutations. J Clin Endocrinol Metab (2021) 106:1183–94. doi: 10.1210/clinem/dgaa749 PMC7993578 33106857

[22] Zheng K Liu T Zhao J Meng P Bian Y Ni C. Mutational Landscape and Potential Therapeutic Targets for Sporadic Pancreatic Neuroendocrine Tumors Based on Target Next- Generation Sequencing. Exp Ther Med (2021) 21:415. doi: 10.3892/etm.2021.9859 33747156 PMC7967861

[23] Comino-Méndez I Tejera ÁM Currás-Freixes M Remacha L Gonzalvo P Tonda R. ATRX Driver Mutation in a Composite Malignant Pheochromocytoma. Cancer Genet (2016) 209:272–7. doi: 10.1016/j.cancergen.2016.04.058 27209355

[24] Zhang J Jiang J Luo Y Li X Lu Z Liu Y. Molecular Evaluation of a Sporadic Paraganglioma With Concurrent IDH1 and ATRX Mutations. Endocrine (2018) 61:216–23. doi: 10.1007/s12020-018-1617-1 PMC7461619 29846902

[25] Luchetti A Walsh D Rodger F Clark G Martin T Irving R. Profiling of Somatic Mutations in Phaeochromocytoma and Paraganglioma by Targeted Next Generation Sequencing Analysis. Int J Endocrinol (2015) 2015:138573. doi: 10.1155/2015/138573 25883647 PMC4390106

[26] Gniado E Carracher CP Sharma S. Simultaneous Occurrence of Germline Mutations of SDHB and TP53 in a Patient With Metastatic Pheochromocytoma. J Clin Endocrinol Metab (2020) 105:dgz269. doi: 10.1210/clinem/dgz269 31851316

[27] Antonio K Valdez MMN Mercado-Asis L Taïeb D Pacak K. Pheochromocytoma/paraganglioma: Recent Updates in Genetics, Biochemistry, Immunohistochemistry, Metabolomics, Imaging and Therapeutic Options. Gland Surg (2020) 9:105–23. doi: 10.21037/gs.2019.10.25 PMC7082276 32206603

[28] Lenders JWM Kerstens MN Amar L Prejbisz A Robledo M Taieb D. Genetics, Diagnosis, Management and Future Directions of Research of Phaeochromocytoma and Paraganglioma: A Position Statement and Consensus of the Working Group on Endocrine Hypertension of the European Society of Hypertension. J Hypertens (2020) 38:1443–56. doi: 10.1097/HJH.0000000000002438 PMC7486815 32412940

[29] Jha A Patel M Baker E Gonzales KM Ling A Millo C. Role of 68Ga-DOTATATE PET/CT in a Case of *SDHB-*Related Pterygopalatine Fossa Paraganglioma Successfully Controlled With Octreotide. Nucl Med Mol Imaging (2020) 54(1):48–52. doi: 10.1007/s13139-019-00629-3 32206131 PMC7062954

[30] Valentijn LJ Koster J Zwijnenburg DA Hasselt NE van Sluis P Volckmann R. TERT Rearrangements Are Frequent in Neuroblastoma and Identify Aggressive Tumors. Nat Genet (2015) 47:1411–4. doi: 10.1038/ng.3438 26523776

[31] Taïeb D Jha A Treglia G Pacak K. Molecular Imaging and Radionuclide Therapy of Pheochromocytoma and Paraganglioma in the Era of Genomic Characterization of Disease Subgroups. Endocr Relat Cancer (2019) 26(11):627–52. doi: 10.1530/ERC-19-0165 PMC7002202 31561209

[32] Carrasquillo AJ Chen CC Jha A Ling A Lin IF Daniel A. Imaging of Pheochromocytoma and Paraganglioma. J Nucl Med (2021) 62(8):1033–42. doi: 10.2967/jnumed.120.259689 PMC8833868 34330739

[33] Taïeb D Hicks JR Hindié E Guillet AB Avram A Ghedini P. European Association of Nuclear Medicine Practice Guideline/Society of Nuclear Medicine and Molecular Imaging Procedure Standard 2019 for Radionuclide Imaging of Phaeochromocytoma and Paraganglioma. Eur J Nucl Med Mol Imaging (2019) 46(10):2112–37. doi: 10.1007/s00259-019-04398-1 PMC7446938 31254038

