# Supplementary material for: Case Report: Malignant Primary Sellar Paraganglioma With Unusual Genetic and Imaging Features
Source: Front Oncol. 2021 Nov 23;11:739255. doi: 10.3389/fonc.2021.739255 (PMC8650633; doi:10.3389/fonc.2021.739255)
Supplement: Supplementary file 1 [file DataSheet_1.docx]

Supplementary Material

# Supplementary Figures and Tables

## Supplementary Figures


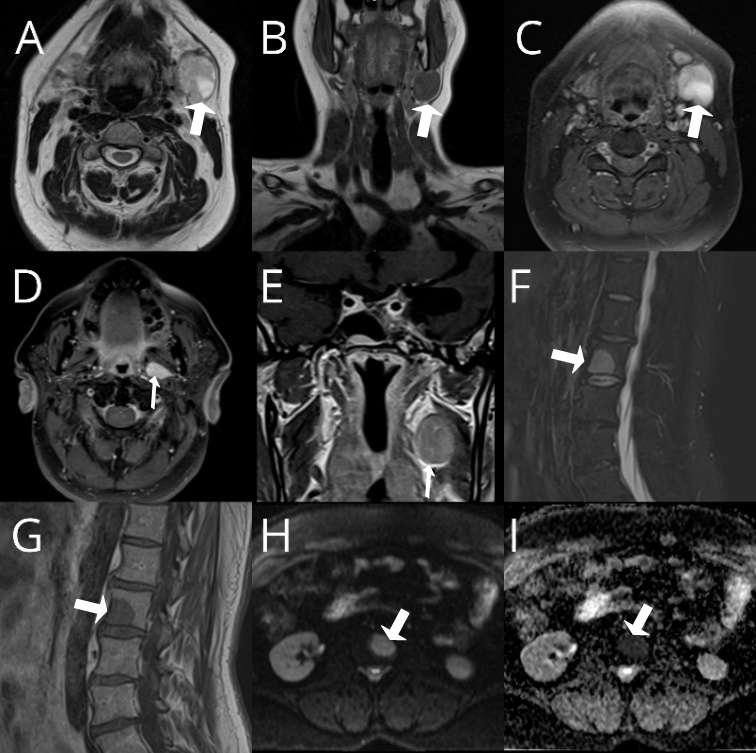


**Supplementary Figure 1.** T2W axial (A), T1W coronal (B), T1W FS axial after contrast administration (C and D) and T1W coronal after contrast administration (E) showing soft tissue nodular lesion with necrotic component in the left submandibular region (thick arrow), and also smaller nodular neoplastic lesion in the masticator space (thin arrow) on the same side. STIR sagittal (F), T1W sagittal (G), DWI (H) and ADC map (I) showing soft tissue lesion (arrows) in the second lumbar vertebra consisted with metastatic lesion.


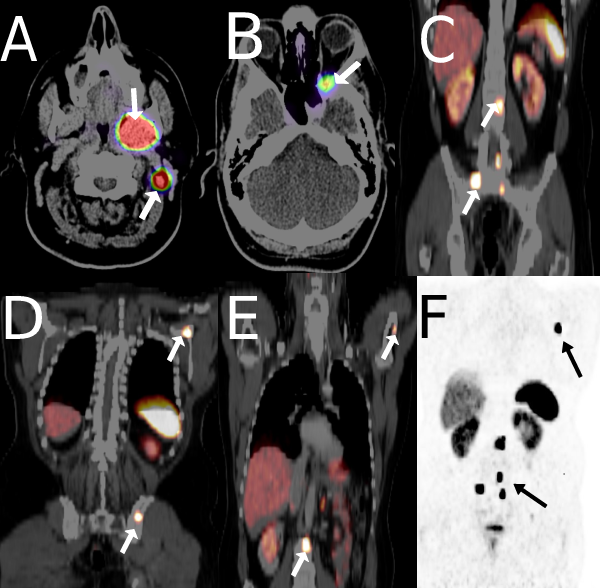


**Supplementary Figure 2.** ^68^Ga DOTA-NOC PET/CT showing the full extent of the disease. Multiple foci of increased activity and radiotracer uptake were found in: left masticator and jugular space (A), left orbit (B), lumbar vertebral bodies and right sacroiliac joint (C), left iliac bone, left scapula (D) and left humerus (indicating by arrows). Maximal intensity projection (MIP) image (F) showing multiple foci of increased activity and radiotracer uptake in lumbar vertebral bodies and left scapula consisted with bone metastasis (indicated by black arrows).

## Supplementary Table

**Supplementary Table 1.** Results of endocrinological examination

|  | **Hormone measurements** | | | | | |
| --- | --- | --- | --- | --- | --- | --- |
| **VARIABLES** | 2008  November before 1^st^ surgery | 2009  April after 1^st^ surgery | 2013  December after radiosurgery | 2015  two years after radiosurgery | 2018  after 2^nd^ surgery | normal  range |
| FT4 (pmol/l) | 7.9 | 8.5 | 13.0 | 12.6 | 14.3 | 9.0-19.0 |
| FT3 (pmol/l) | 3.2 | 3.7 | 4.6 | - | - | 2.6-5.7 |
| TSH (mIU/l) | 1.26 | 0.13 | - | 0.01 | - | 0.35-4.95 |
| GH (ng/ml) | 0.33 | - | - | - | - | <2.5 |
| IGF 1 (ng/ml) | - | 64.0 | - | - | - | 109.0-284.0 |
| ACTH (pg/ml) | 28.0 | 23.0 | 18.0 | 10.0 | - | 7.0-63.0 |
| CORT (nmol/l) 8am | 315.0 | 228.0 | 400.0 | <13.8 | - | 150-620 |
| CORT (nmol/l) 6pm | 158.0 | 78.0 | 130.4 | <13.0 | - | 85-410 |
| Synacthen CORT 0’ | - | 240.0 | - | - | - | - |
| Synacthen CORT 30’ | - | 773.0 | - | - | - | >500.0 |
| Synacthen CORT 60’ | - | 943.0 | - | - | - | - |
| PRL 8am | 1.4 | 0.75 | <0.6 | - | - | 3.5-19.4 |
| PRL 11am | - | - | <0.6 | - | - |  |
| FSH (IU/l) | 2.6 | 0.5 | 1.1 | - | - | 0.9-7.0 |
| LH (IU/l) | 2.2 | 0.6 | 1.2 | - | - | 1.4-7.7 |
| Testosterone (nmol/l) | 12.9 | 7.99 | 8.51 | - | - | 8.33-30.19 |
| Osm P (mOsm/kg) | 291.0 | - | 296.0 | - | 292.0 | 270-300 |
| Osm U (mOsm/kg) | 376.0 | - | 573.0 | - | 511.0 | 30-1300 |
| Metanephrin (pg/ml) | - | - | - | - | <36.0 | <65.0 |
| Normetanephrin (pg/ml) | - | - | - | - | 180.0 | <196.0 |

FT3 - free triiodothyronine ; FT4 - free thyroxine; TSH – thyroid stimulating hormone; CORT – cortisol; ACTH – adrenocorticotropic hormone; GH – growth hormone; IGF 1 – insulin-like growth factor 1; PRL – prolactin; FSH – Follicle stimulating hormone; LH – luteinizing hormone; Osm P – plasma osmolality; Osm U – urine osmolality
